# Editorial: Light-Assisted Molecular and Hybrid Systems for Artificial Photosynthesis

**DOI:** 10.3389/fchem.2022.868373

**Published:** 2022-04-07

**Authors:** Mirco Natali, Xavier Sala, Souvik Roy, Andrea Pannwitz, Albert Ruggi

**Affiliations:** ^1^ Department of Chemical, Pharmaceutical and Agricultural Sciences (DOCPAS), University of Ferrara, Ferrara, Italy; ^2^ Departament de Química, Unitat de Química Inorgànica, Universitat Autònoma de Barcelona, Barcelona, Spain; ^3^ Joseph Banks Laboratories, School of Chemistry, University of Lincoln, Lincoln, United Kingdom; ^4^ Institute of Inorganic Chemistry I, Ulm University, Ulm, Germany; ^5^ Département de Chimie, Université de Fribourg, Fribourg, Switzerland

**Keywords:** artificial photosynthesis, molecular catalyst, hybrid system, photoelectrode, energy, photosensitizer

The rapid increase in the global energy demand in combination with the greenhouse gas effects of traditional fossil fuels calls for the development of green and renewable energy resources worldwide. The quest for cost-effective technologies to harvest renewable energy sources and the production of carbon-neutral fuels are thus of paramount importance in the framework of sustainable development (Lewis and Nocera, 2006; Gray, 2009). Sunlight constitutes perhaps the most attractive renewable source of energy, considering its wide availability and its immense energy flux (in the order of 1 kW/m^2^). However, from a practical standpoint, direct utilization of sunlight is often unfeasible due to its intrinsic intermittency and fluctuating intensity (Balzani et al., 2008). In this regard, conversion of solar energy into chemical energy, termed “Artificial Photosynthesis” (AP), represents a viable strategy that takes inspiration from the natural light-driven reactions occurring in green plants and bacteria (Dau et al., 2017). Depending on the design of the respective AP system, the generated product can be directly used as feedstock for industrial processes or as fuel.

A basic set of components for light energy conversion into chemical energy carriers are the following: (i) a photosensitizer, which is responsible for light absorption and charge separation, and (ii) catalytic units, capable of storing photogenerated electrons and holes to facilitate the conversion of substrates into products *via* multi-electron/multi-proton processes. Based upon the nature of the active components, AP systems can be classed into molecular or material-based (Hisatomi et al., 2014; Andreiadis et al., 2011). Molecular species have a defined structure, which enables in-depth mechanistic studies and fine-tuning of electronic properties *via* chemical modifications. Conversely, solid-state materials exhibit higher stability and durability but are limited in terms of tunability and selectivity. *Hybrid systems* aim at merging the *best of both strategies* (Zhang and Sun, 2019; Smith et al., 2020)*.*

Different reaction schemes can be designed depending on the chemical feedstock or fuel to be produced by the reductive half-reaction. All these processes require electrons which should ideally be provided from a parallel oxidative process in an electronically coupled anodic compartment (Wang et al., 2021). In this research topic, several approaches for both oxidative and reductive light energy conversion are covered. Specifically, we proudly present two publications on the oxidative half-reaction, and three publications covering the reductive half-reactions, including CO_2_ reduction and H_2_ evolution.

To shed light on the new developments in the field of hybrid materials for the oxidation half-reaction, Alemán and Mas-Ballesté have reviewed the application of Covalent Organic Frameworks (COFs) and Covalent Triazine Frameworks (CTFs) in photocatalytic oxidation processes comprising oxidation of organic substrates such as alcohols, sulfides, and amines, as well as water oxidation to dioxygen when coupled with suitable metal oxides as co-catalysts. The effects of porosity and crystallinity on the final photocatalytic activity have been critically evaluated, highlighting the potential and future challenges for the design and practical application of these interesting organic-based reticular materials.

Although the choice of the light-harvesting components and catalytic units are usually of fundamental importance for water oxidation in hybrid photoanodes, semiconductor support also plays a key role. This challenge has been addressed by Gong et al. who constructed a novel photoanode material for the water oxidation reaction by replacing conventional mesoporous TiO_2_ with anatase-wrapped arrays of single-crystal TiO_2_ rutile nanorods (ARNRs). Functionalization with polymeric carbon nitride as the sensitizing unit and CoO(OH)_x_ as the catalyst turned out to be effective in promoting visible-light-driven oxygen evolution. The promising performance of ARNRs as large surface area electron-collector in hybrid photoanodes based on polymeric light-harvesting materials is expected to stimulate further efforts in the field of photoelectrochemistry for solar fuels.

On the reductive side of AP, CO_2_ reduction currently represents one of the most important conversions, as it combines the possibilities of generating a fuel (or chemical feedstock) with the control over the CO_2_ level in the atmosphere. A major challenge in CO_2_ reduction is steering the selectivity towards carbon-based products while suppressing the competitive H_2_ evolution reaction. Obermeier et al. have reported the synthesis of novel molecular catalysts based on Earth-abundant 3d transition metals (Fe, Co, Ni) with pentadentate nitrogen and sulfur-chelating ligands and their application under light-driven conditions with copper and iridium complexes as light-harvesting photosensitizers and triethylamine as the sacrificial electron donor. By varying the choice of metal and ligand of the catalyst as well as the photosensitizer the authors have identified conditions to control the product selectivity towards either CO, H_2_, or a mixture of both. These results thus point out how modification of molecular catalysts through chemical synthesis represents a valuable tool to fine-tune the selectivity of (photo)catalytic small molecule activation.

Addressing the role of directly using CO_2_ as a feedstock for organic synthesis, Franceschi et al. have provided a thermodynamic analysis guided by DFT computations to predict the reactivity of carbanions with CO_2_ based on the acidity of the CH/C^−^ couple, which is relevant for reactions such as photo- or electro-driven carboxylations. Interestingly, the predicted reactivity trend has been substantiated with electrochemical studies involving, among others, flavone and chalcone as model compounds, thereby proving the versatility of the presented method to target novel reactivity patterns aimed at the fixation of CO_2_ into organic scaffolds.

In the context of hybrid electrodes for proton or CO_2_ reduction Cerpentier et al. report on the synthesis of two supramolecular dyads that combine a ruthenium polypyridine photosensitizer with either a rhenium catalyst for light-driven CO_2_ reduction or a platinum catalyst for H_2_ production. Bearing suitable anchoring groups on the ruthenium polypyridine unit enabled immobilization onto mesoporous NiO electrodes and thereby the construction of photocathodes for CO_2_ or proton reduction, respectively. Differences in reactivity suggest differences in mechanism between operation in homogeneous solution and on NiO. Time-resolved infrared spectroscopy and spectroelectrochemistry methods finally provide important insights for the construction of active photocathodes.

Overall, the set of papers collected represents cutting-edge scientific reports in a very important research field, which are expected to stimulate scientific discussions and novel ideas to target the critical, global challenge of developing new, practical, and cost-efficient renewable energy technologies.
